# Conservation Genetics of a Critically Endangered Limpet Genus and Rediscovery of an Extinct Species

**DOI:** 10.1371/journal.pone.0020496

**Published:** 2011-05-31

**Authors:** Diarmaid Ó Foighil, Jingchun Li, Taehwan Lee, Paul Johnson, Ryan Evans, John B. Burch

**Affiliations:** 1 Museum of Zoology and Department of Ecology and Evolutionary Biology, The University of Michigan, Ann Arbor, Michigan, United States of America; 2 Alabama Aquatic Biodiversity Center, Marion, Alabama, United States of America; 3 Kentucky State Nature Preserves Commission, Frankfort, Kentucky, United States of America; Zoological Society of London, United Kingdom

## Abstract

**Background:**

A third of all known freshwater mollusk extinctions worldwide have occurred within a single medium-sized American drainage. The Mobile River Basin (MRB) of Alabama, a global hotspot of temperate freshwater biodiversity, was intensively industrialized during the 20^th^ century, driving 47 of its 139 endemic mollusk species to extinction. These include the ancylinid limpet *Rhodacmea filosa*, currently classified as extinct (IUCN Red List), a member of a critically endangered southeastern North American genus reduced to a single known extant population (of *R. elatior*) in the MRB.

**Methodology/Principal Findings:**

We document here the tripling of known extant populations of this North American limpet genus with the rediscovery of enduring *Rhodacmea filosa* in a MRB tributary and of *R. elatior* in its type locality: the Green River, Kentucky, an Ohio River Basin (ORB) tributary. *Rhodacmea* species are diagnosed using untested conchological traits and we reassessed their systematic and conservation status across both basins using morphometric and genetic characters. Our data corroborated the taxonomic validity of *Rhodacmea filosa* and we inferred a within-MRB cladogenic origin from a common ancestor bearing the *R*. *elatior* shell phenotype. The geographically-isolated MRB and ORB *R*. *elatior* populations formed a cryptic species complex: although overlapping morphometrically, they exhibited a pronounced phylogenetic disjunction that greatly exceeded that of within-MRB *R*. *elatior* and *R. filosa* sister species.

**Conclusions/Significance:**

*Rhodacmea filosa*, the type species of the genus, is not extinct. It persists in a Coosa River tributary and morphometric and phylogenetic analyses confirm its taxonomic validity. All three surviving populations of the genus *Rhodacmea* merit specific status. They collectively contain all known survivors of a phylogenetically highly distinctive North American endemic genus and therefore represent a concentrated fraction of continental freshwater gastropod biodiversity. We recommend the establishment of a proactive targeted conservation program that may include their captive propagation and reintroduction.

## Introduction

Surface freshwater habitats support a disproportionate share of the earth's biodiversity: they embody 0.01% of the planet's water [1] but contain >9% of described animal species, including one third of vertebrates [2]. They also represent a critical resource for a burgeoning human population and approximately 65% of global river discharge is currently under moderate to high anthropogenic threat [3]. Throughout history, human water security needs have routinely superseded biodiversity concerns [3] and an estimated 10,000–20,000 freshwater species have been imperiled or made extinct through human agency, including over a third of many taxonomic groups in the heavily industrialized watersheds of North America and Europe [4], [5].

**Figure 1:**
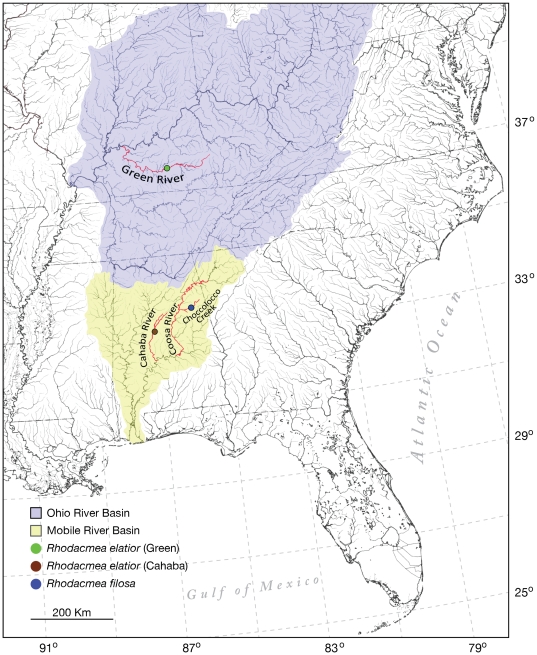
Map of southeastern U.S.A. watersheds. The Ohio and Mobile River Basins are highlighted to show the locations of all known extant *Rhodacmea elatior* and *R. filosa* populations.

North American drainages experienced a major wave of impoundment during the early-mid 20^th^ century and <2% (<100,000 km) of high-quality, free-flowing rivers remain of an estimated original U.S. figure of 5,200,000 km [6]. Damming completely transforms dynamic lotic habitats, turning free-flowing rivers into chains of reservoirs with severe consequences for endemic biotas [7]. This is most starkly evident in the southeastern United States Mobile River Basin (MRB; Fig. 1), a global hotspot of temperate freshwater fish and mollusk diversity characterized by extraordinary levels of endemism, including 40 fish species [8] and 34 mussel and 105 snail species [9], [10] found nowhere else. The MRB was transformed by the construction of 36 major dams and locks that replaced endemic species-rich riverine shoal, riffle and pool habitats with lentic reservoirs along much of the mainstem MRB rivers and their tributary streams [11]. Its endemic malacofauna was especially hard hit and an estimated 10 mussel species and 37 snail species were driven to extinction ([11]–[13], PJ, unpubl.). This represents a third of all known freshwater molluscan extinctions worldwide [14] and has been referred to as “one of the greatest known extinction episodes of the first half of the twentieth century” [15].

One of these losses involved a MRB endemic limpet, the wicker ancylinid, *Rhodacmea filosa* (Conrad, 1834), that occurred in MRB rivers and their tributaries [16] but was formally declared extinct (IUCN Red List) in 2000 [5]. The North American freshwater limpet genus *Rhodacmea* Walker, 1917 is endemic to fast-flowing Southern Appalachian rivers and streams feeding the MRB and Ohio River drainages (Fig. 1). It is composed of three nominal species [*R. filosa*; *R. elatior* (Anthony, 1855); *R. hinkleyi* (Walker, 1908)], distinguished solely by shell phenotype characteristics (see Materials and Methods), but the last taxonomic revision contained the caveat that all three “may represent ecological variants of a single relatively unstable phylogenetic twig” [16]. The genus was very severely impacted by early 20^th^ century watershed industrialization and subsequently only one extant population – of *R. elatior* in the Cahaba River segment of the MRB – was known [16]–[18]. Although its extreme rarity has hindered recent study, the anatomical distinctiveness of the genus *Rhodacmea* has long been apparent and in some taxonomic schemes it was placed in its own sub-family [19], [20] or family [21]. Other workers proposed a sister relationship with the Eurasian freshwater limpet genus *Ancylus* [22], [23] and this interpretation has been corroborated (weakly) by recent molecular phylogenetic characterization of the surviving Cahaba *R. elatior* population [24].

It can be extraordinarily difficult to establish with certainty the extinction status of critically endangered species in nature [25], as exemplified by the controversy surrounding the purported rediscovery of the ivory-billed woodpecker (*Campephilus principalis*) [26]. An important subtext to the MRB biodiversity crisis is that improved water management and anti-pollution policies over the last three decades have led to significant recovery and expansion of surviving molluscan endemics in the remaining unimpounded segments of the drainage [27]. This resurgence, together with greatly expanded field surveying efforts by multiple agencies [27]–[30], have led to the rediscovery of a number of persistent populations of endemic species previously thought to have been extirpated [31]–[33].

Recently, one of the authors (PJ) encountered a surviving population of putative *Rhodacmea filosa* in an MRB tributary and another (RE) detected a second population of *R. elatior* external to the MRB in its type locality, the Green River, KY, an Ohio River tributary. These new discoveries tripled the known populations of this phylogentically distinctive North American genus and offered us a hitherto unimaginable opportunity to reassess its conservation status across multiple basins using morphometric and genetic characters. This is important because *Rhodacmea* species have been diagnosed solely on conchological traits that may well be ecophenotypic [16], recent studies of two other North American ancylid limpet genera have prompted considerable taxonomic rationalization [24], [34], and valid species designations are prerequisites for sound conservation planning [35]. Our results confirm that *R. filosa* is extant, validate its specific status on morphometric and phylogenetic grounds, and show that all three surviving populations of this genus represent distinct evolutionary lineages that merit specific status.

## Results

**Figure 2:**
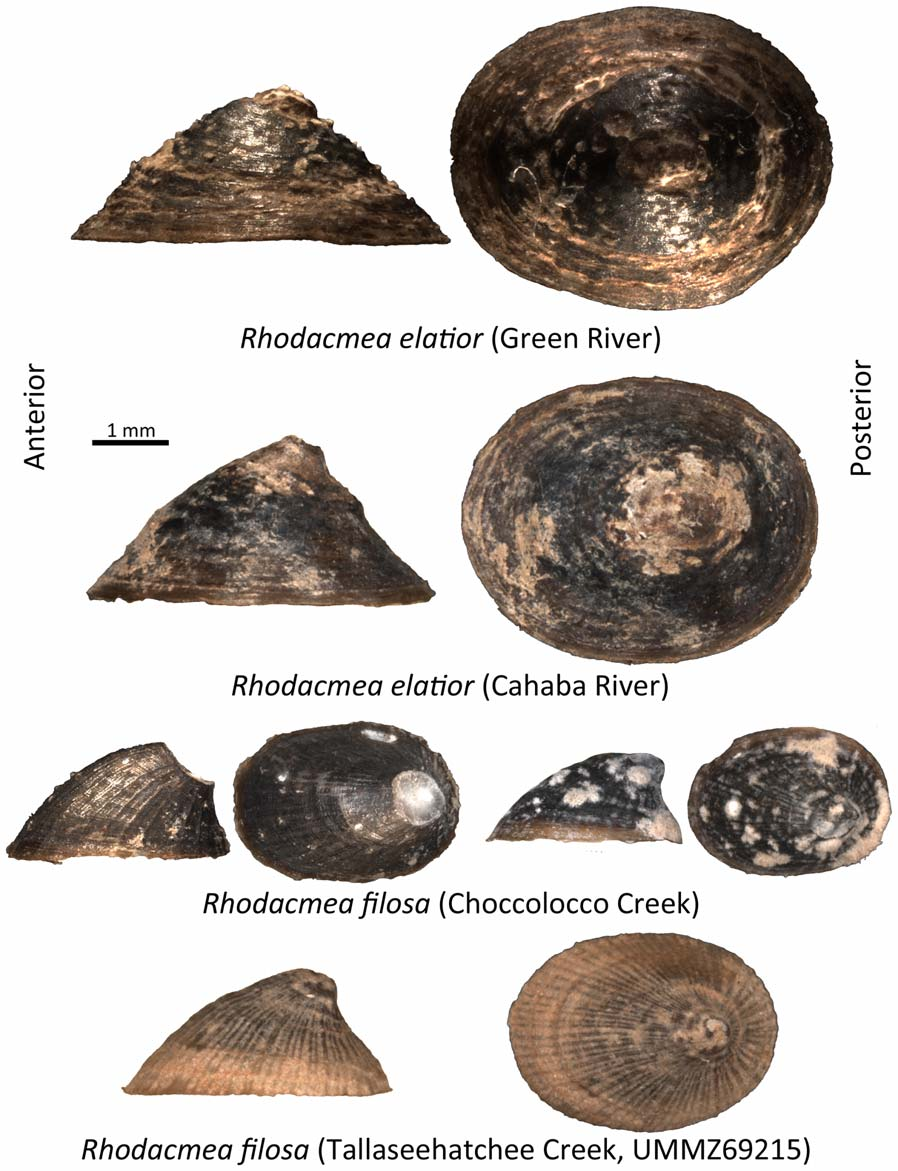
Left-lateral and dorsal views of exemplar *Rhodacmea spp.* shells. Specimens from both extant *Rhodacmea elatior* populations (Green River; Cahaba River) are shown. Two specimens from the single extant *R. filosa* population (Choccolocco Creek, the individual on the left has lost its apex) are also depicted, in addition to a century old museum exemplar (Tallaseehatchee Creek; UMMZ 69215).

Figure 2 shows lateral and dorsal views of exemplar limpets sampled from all three extant populations (Fig. 1) together with a century old museum reference specimen of *Rhodacmea filosa* (UMMZ 69215) from Tallaseehatchee Creek, another Coosa tributary. Green River and Cahaba River limpets all exhibited diagnostic *R. elatior* conchological features: elevated patelliform shells that lack ribbing and have clearly convex anterior slopes [16], [36]. Limpets from Choccolocco Creek, a Coosa River tributary, were much smaller and all unambiguously displayed the diagnostic feature of *R. filosa*: ribbing in the form of strong radiating lines running from the apex to the aperture [16], [37]. None of the limpets matched the description of *R. hinkleyi* (Walker, 1908), the third nominal member of this genus [16].

**Figure 3:**
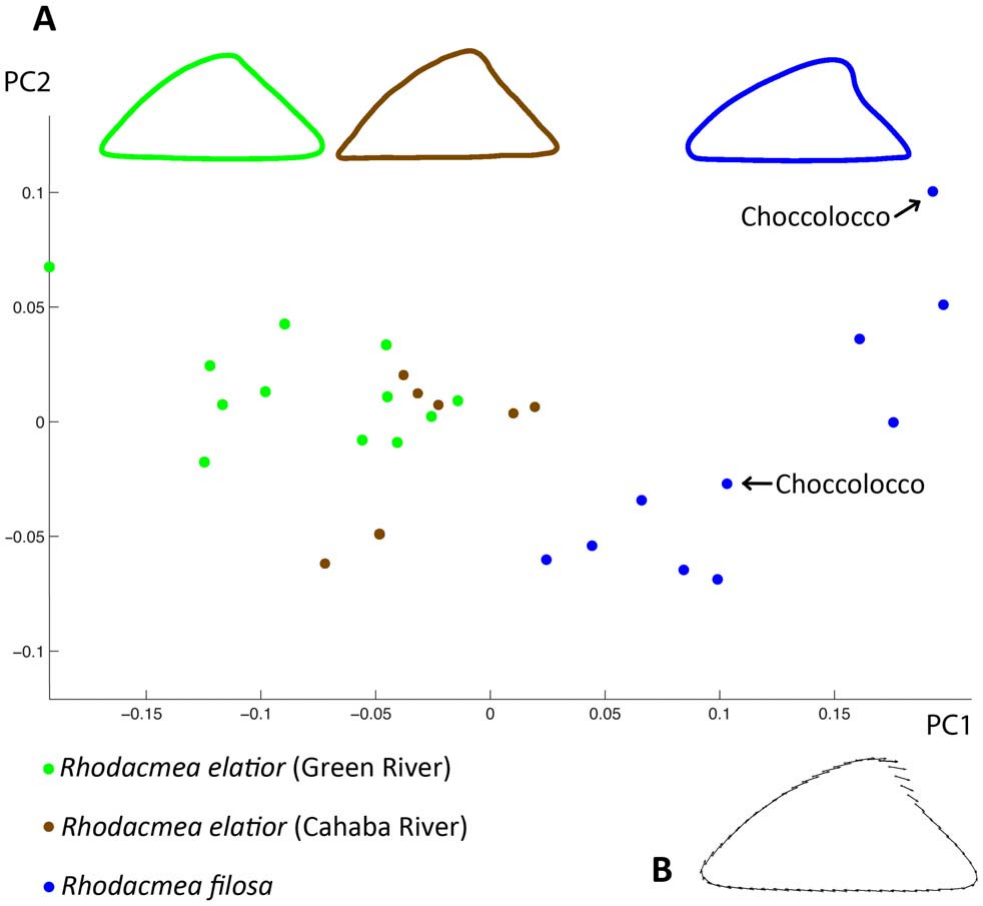
Morphometric analyses of *Rhodacmea* populations. 3A. Scatter plot showing shell shape principal component analyses of individual limpets from three groups: 1) Green River *Rhodacmea elatior* (*n* = 12); 2) Cahaba River *R. elatior* (*n* = 7); 3) a combined sample of apically-intact *R. filosa* from an extant Choccolocco Creek population (*n* = 2, arrowed) and museum specimens from an extirpated Tallaseehatchee Creek population (*n* = 8). The mean shapes of each group are shown at the top of the plot. 3B. A composite outline showing PCA deformation vectors on individual landmarks for PC1 representing changes between a consensus shape for all limpets analysed (PC1 = 0) and that of a hypothetical limpet positioned mid-range (PC1 = 0.1) for *Rhodacmea filosa.*

In all but two of the Choccolocco Creek *Rhodacmea filosa* specimens sampled, the shell apex had broken cleanly off leaving an abruptly truncated apical profile (Fig. 2). Availability of only two apically-intact exemplars complicated our morphometric analyses and we compensated for this by including intact museum Tallaseehatchee Creek reference specimens of *R. filosa* (UMMZ 69215) (Fig. 2). Figure 3 depicts a principal component analysis plot for limpet lateral profiles sampled from all three extant populations (Fig. 1) as well as from the *R. filosa* museum sample. The first principal component (PC1) explained 80% of variation in shell shape among all of the limpets. Green River and Cahaba River *R. elatior* populations overlapped for both PC1 and PC2, as did the extant Choccolocco and extirpated Tallaseehatchee *R. filosa* populations. However, *R. elatior* specimens were well separated from *R. filosa* specimens along PC1. As shown by the PCA deformation graph (Fig. 3B), the most pronounced morphometric difference between the two species occurred in the apical region where intact *R. filosa* specimens possessed a posteriorally-projecting apex (a feature mentioned in the original species description [36], [37]) absent in *R. elatior* (Figs. 2, 3B).

**Figure 4:**
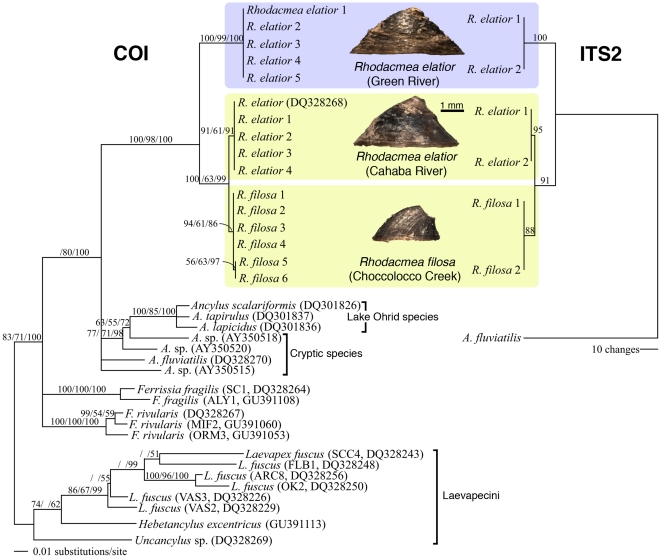
Molecular phylogenetic analyses of *Rhodacmea* populations. Results for two genetic markers are shown: a bayesian consensus phylogram derived from the mitochondrial cytochrome oxidase I (mt COI) dataset and the single most parsimonious tree found for the nuclear second internal transcribed ribosomal spacer (ITS-2) dataset. For the former, available New World Laevapecini haplotypes were employed as outgroups [24]. For the latter, the Old World *Ancylus fluviatilis* was the outgroup [24]. Nodal support levels >50 are shown above the respective branches: MP bootstrap/ML bootstrap/Bayesian posterior probabilities for COI tree and MP bootstrap for ITS-2 tree. GenBank accession numbers are given for non-novel genotypes.

Limpets from all three study populations were genotyped for three gene fragments. One of these, a relatively conserved large nuclear ribosomal (28S rDNA) gene, was monomorphic across all three populations. They exhibited the same 28S genotype published previously for Cahaba *R. elatior* [24] that placed sister to the European ancylid *Ancylus fluviatilis* in global ancylinid 28S gene trees [24], [34]. The other two gene fragments exhibited population-level genetic differences and the results of their phylogenetic analyses are presented in Fig. 4. For the mitochondrial marker (COI), the availability of an extensive ancylinid GenBank database allowed us to place the *Rhodacmea* lineages within the Holartic Ancylinae, using the New World Laevapecini as outgroups [24]. Our mt COI topologies corrorborated the phylogenetic distinctiveness of the genus *Rhodacmea* within North America [24], [34], showing it to be sister to the western Palaeartic genus *Ancylus*, rather than to the other North American ancylinid genera (*Ferrissia*, *Laevapex* and *Hebetancylus*).

Unlike the morphometric analysis (Fig. 3), our mitochondrial phylogenies (Fig. 4) consistently parsed the three extant *Rhodacmea* populations on the basis of river basin (Ohio/Mobile) rather than taxonomy (*R. elatior/R. filosa*). A pronounced (>5.5% mt COI uncorrected sequence divergence) and phylogenetically robust genetic disjunction separated the Green River *R. elatior* from MRB limpets of either species. MRB *R*. *elatior* and *R. filosa* haplotypes formed a phylogenetically-robust, but shallow (<1.0% mt COI uncorrected sequence divergence), clade in which the two species were reciprocally monophyletic (Fig. 4). This result was corroborated when limpets from the three populations were cross-referenced with nuclear ribosomal second internal transcribed spacer (ITS-2) genotypes. Using *Ancylus fluviatilis* as an outgroup, Green River *R. elatior* were robustly sister to MRB limpets of either species due to the presence of multiple basin-specific nucleotide substitutions (Fig. 4). Within the MRB, *R*. *elatior* and *R. filosa* ITS-2 genotypes differed only by a single 8-nucleotide insertion/deletion. In parsimony analyses with gaps coded as newstates, this insertion was sufficient to render MRB *R*. *elatior* and *R. filosa* reciprocally monophyletic (Fig. 4).

## Discussion

Our primary finding is that the officially extinct [5] endemic MRB limpet, *Rhodacmea filosa*, the type species of the genus *Rhodacmea*, remains extant in Choccolocco Creek. Its survival there is somewhat surprising, given the serious episodes of industrial pollution experienced by this watershed (see Materials & Methods). Over the past 20 years, extensive surveys, involving hundreds of Coosa, Cahaba and Black Warrior (its type locality) drainage collecting sites [17], [18], [27]–[29], [38]–[42], have failed to find *R. filosa*. It appears unlikely that multiple additional extant populations await detection outside of Choccolocco Creek.

Although we can confirm that *Rhodacmea filosa* remains extant, the conservation significance of its persistence is potentially undermined [43] by the uncertain taxonomic status of nominal *Rhodacmea* species [16]. Taxonomic uncertainty has long pervaded the North American freshwater limpet literature and it has stemmed from inadequate species-level descriptions [16], [36], [44], compounded by pronounced ecophenotypic plasticity in shell morphology [16], [44]–[46]. Recent phylogenetic analyses have prompted the synonymization of multiple North American nominal species of *Laevapex* [24] and *Ferrissia* [34] and we are now in a position to also test the robustness of *R. filosa* and *R*. *elatior* taxonomic designations.

Our morphometric and molecular phylogenetic data corroborate the taxonomic validity of *Rhodacmea filosa*. The former data (Fig. 3) indicate that this species is distinguishable from *R*. *elatior* on morphometric grounds (in addition to the taxonomically-diagnostic presence of radial ribs) although the distinguishing morphometric feature – a prominent posteriorally-projecting apex – is subject to frequent loss through breakage in the extant population (Fig. 2). The latter data (Fig. 4) demonstrate that within-MRB reciprocal monophyly of these taxa for both mitochondrial and nuclear markers has been achieved. *R. filosa* therefore possesses two major attributes of a well-established species: fixed morphological apomorphies and gene tree monophyly [47]. Our phylogenetic data are also consistent with a within-MRB cladogenic origin of *R. filosa* from a common ancestor bearing the *R*. *elatior* shell phenotype (Fig. 4) and this highlights the importance of the MRB as a regional cradle of freshwater diversification in North America. *Rhodacmea*'s Old World sister genus, *Ancylus* (Fig. 4), has undergone a parallel speciation process in a Balkan ancient lake (Lake Ohrid) yielding three morphologically-differentiated endemic species, *A. tapirulus*, *A. lapicidus* and *A. scalariformis* [48] that also exhibit modest levels of mitochondrial genetic divergence (Fig. 4).

Morphometric and molecular phylogenetic analysis of *Rhodacmea elatior* yielded divergent signals concerning the inter-relationships of nominally conspecific Green River (Ohio Basin) and Cahaba River (MRB) populations. They overlapped substantially in morphospace (Fig. 3), a result congruent with Basch's inability to separate Anthony's 1855 Green River specimens from Cahaba limpets [16], although quantitative analyses of larger sample sizes might well reveal statistical differences among the two populations. However, extant Green and Cahaba river populations proved to be highly differentiated genetically, much more so than within-MRB *R*. *elatior* and *R. filosa* sister lineages, and they were reciprocally monophyletic in mitochondrial and in nuclear gene trees (Fig. 4). *R*. *elatior*'s pronounced, but cryptic, among-basin phylogenetic disjunction was unexpected: three other ancylinids (*Laevapex fuscus*, *Ferrissia rivularis* and *F. fragilis*) showed no evidence of such across Eastern North American drainages [24], [34], although estimates of inter-populational gene flow for *L. fuscus* were generally low at both regional and continental scales [24]. Among-drainage migration events by freshwater limpets are most likely achieved *via* phoresy [49] and it is possible that *R*. *elatior*'s inter-basin phylogenetic disjunction reflects a lowered affinity for this method of dispersal relative to other North American ancylinids.

*Rhodacmea elatior*'s combination of morphological conservatism and cryptic among-basin phylogenetic structuring does have parallels in its Old World sister genus, *Ancylus* (Fig. 4). Four of eight phylogenetically-distinct species of *Ancylus* [48] form a cryptic species complex with largely allopatric distributions [50]. Although the four cryptic species can be (*a posteriori*) differentiated morphometrically, morphological differentiation is not feasible without molecular genotyping [50]. Only one, *A. fluvialis*, bears a formal species name, but all four are viewed as having speciated, *i.e.*, formed reproductively and genetically isolated lineages [50].

What then of Green River and Cahaba River *Rhodacmea elatior* – have they also speciated? This is a non-trivial question in terms of conservation biology status (e.g., the U.S. Endangered Species Act) and the issue is compounded by the plethora of competing species definitions that hinder the emergence of standardized methodologies [35], [43], [51]–[53]. Nevertheless, two salient conclusions can be made concerning these limpet populations. Firstly, restriction of these sedentary, direct-developing mollusks to the mountain headwaters of two different major basins means that they have no direct possibility of exchanging genes. They therefore fall outside the frame of reference of the biological species concept [54]. Secondly, this reproductive isolation is of sufficiently long standing to have generated pronounced reciprocal monophyly for both nuclear and mitochondrial markers (Fig. 4). This condition exceeds the within-species category of “evolutionary significant units” (*sensu* [55]), but complies with a diagnosis of speciation according to the phylogenetic species concept [56].

The Green/Cahaba *R*. *elatior* phylogenetic disjunction approximates that of cryptic European *Ancylus* species (Fig. 4) that have been tentatively dated (using a range of sequence divergence rates) from 0.9–7.8 Ma [50]. However, it is clearly much older than that of within-MRB *R*. *elatior* and *R. filosa* sister lineages (Fig. 4) that we have judged to have speciated. This last detail, together with the unambiguous phylogenetic evidence that Green and Cahaba populations represent separate evolutionary trajectories (Fig. 4), lead us to propose that they have cryptically speciated and to recommend that their status be formally revised in a subsequent taxonomic publication: *R*. *elatior* for Green River limpets and (presumably) *R. cahawbensis* Walker, 1917 for Cahaba River limpets.

The three extant limpet populations analysed in this study represent a highly concentrated fraction of North American freshwater gastropod biodiversity, irrespective of whether that is calculated in terms of species richness [57] or of phylogenetic diversity [58]. Our data indicate that each extant population represents the only known living representatives of a discrete species: two members of a *Rhodacmea elatior* cryptic species complex and *R. filosa*. There is, to our knowledge, no record of extant *R. hinckleyi*. Our three study poplations therefore contain all known extant members of a phylogentically highly distinctive North American endemic genus. Nominally extinct taxa occasionally persist in the wild, to be rediscovered at a later date [59], but in many such cases this rediscovery is tempered by the reality that, at best, the relevant species are nearly extinct [60]. The “nearly extinct” label clearly applies to all three inferred *Rhodacmea* taxa and the long-term difficulty of preserving freshwater biodiversity, even in wealthy countries with significant conservation programs [3], highlights the formidable nature of the conservation challenge. Nevertheless, there has been a major renaissance in the conservation biology of freshwater North American mollusks in recent years, exemplified by the establishment of the *Freshwater Mollusk Conservation Society* in 1998, and the establishment of a large-scale program of captive breeding and reintroduction by the Alabama Aquatic Biodiversity Center in 2006. The extensive evolutionary history retained within the three surviving *Rhodacmea* populations imbues them with exceptionally high biodiversity value, thereby making their conservation maximally cost-effective [61] and justifying a proactive program of protection, propagation and reintroduction.

## Materials and Methods

### Specimen Collecting

Alabama and Kentucky state scientific collection permits were obtained prior to sampling. Given the extreme rarity of *Rhodacmea* species, conservation concerns led us to limit our sampling to ≤10 individuals each in the two MRB locations and to 13 specimens in the Green River population. This was sufficient to taxonomically identify the limpets, provide voucher specimens and to qualitatively characterize the populations genotypically and morphometrically, but it was insufficient to allow us to perform meaningful quantitative analyses.

The Green River is a 580 km long tributary of the Ohio River and ultimately drains into the Gulf of Mexico via the Mississippi (Fig. 1). It is entirely contained with the state of Kentucky and has a long history of industrialization, starting with partial canalization in 1842 [62]. The Green River is the type locality of *Rhodacmea elatior* but it is notable that in the 1855 species description it was recorded as being “very rare” [63]. In 2010, a targeted search of the type locality at Green River Mile 229 (Munfordville, Hart County), performed by two of the authors (RE & PJ), established the persistence of this population, currently the only know extant record for the Ohio River Basin. Limpets were attached to medium-large cobblestones within 5 m of the bank slope/stream bed interface adjacent to a large shallow (∼20 cm depth) riffle.

The Cahaba River is a 307 km long branch of the MRB in central Alabama (Fig. 1). Although it has not been dammed for hydroelectric power generation and remains the longest free-flowing river in Alabama, it nevertheless lost almost one quarter of its original malacofauna during the 20^th^ century [27]. Species losses were driven by historic point-source discharges and severe bedload disruption in the middle and lower basin. The Cahaba currently contains 31 species of snails, 3 of which are federally protected [27]. Cahaba populations of *Rhodacmea elatior* were originally described by Walker in 1917 as *R. cahawbensis* and he recorded them as occurring on rocks and mussel shells [19]. Surviving populations were documented in Helena, Shelby County in 1959 [16], [22] and at Marvel Slab, Bibb County in 1992 [17], [27]. The latter population remains extant, limpets occur attached to cobble-boulder substrate, within 5 m of the bank/slope in <20 cm of flowing water, but intensive surveying of the Cahaba main stem and tributaries has failed to find additional survivors [17], [27].

Choccolocco Creek is a 91 km long tributary of the Coosa River in central Alabama (Fig. 1). The Coosa is the most heavily impacted major river in the MRB: hydropower dams impound 86% of its mainstem and up to 26 endemic Coosa gastropods have been rendered extinct [11]. Choccolocco Creek has three substantial impoundments as well as a history of severe industrial pollution. Although historic pollution point sources have abated, the creek retains an Alabama Department of Environmental Management “303d” (officially impaired) status for persistent polychlorinated biphenyl (PCB) and mercury contamination. Most of the historic mussel fauna is missing [29] but the creek currently supports more than 25 species of snails (PJ, unpubl.). *Rhodacmea filosa* occurs in the lower portion of the watershed within the highest quality habitat remaining. Specimens were found adjacent to the channel margin attached to cobble-boulder substrate in shallow (<20 cm) flowing water.

### Species Identification

Species identifications were determined using both the original descriptions [36] and the diagnostic conchological characters identified in most recent taxonomic revision of the genus [16]. *Rhodacmea filosa* is the most distinctive, its conspicuous ribbing separating it from the unribbed shells of the others. *R. elatior* has an elevated shell with a clearly convex anterior slope; *R. hinkleyi* is less elevated with a straight or slightly convex anterior slope [16], [36].

### Morphometric Analyses

Limpets from all three localities with extant populations were analyzed, together with apically-intact museum reference specimens of *Rhodacmea filosa* (UMMZ 69215) (Table 1). H. H. Smith sampled the latter specimens during the early-mid 1910's in Tallaseehatchee Creek, another Coosa tributary, parallel to and 35 km south of Choccolocco Creek. He noted that most of the limpets were attached to the shells of living pleurocerid snails, a microhabitat also mentioned in Conrad's original description of this species [37].

**Table 1:** Summary information on the limpet specimens employed in this study.

Taxon	Locality	COI	ITS2	28S	Morphometrics	UMMZ #	GenBank #
*Rhodacmea filosa*	Choccolocco Creek, Talladega Co., Alabama, USA	6	2	2	2	302908	JF304799, 304800, 304803, 304806
	Tallaseehatchee Creek, Talladega Co., Alabama, USA				8	69215	
*Rhodacmea elatior*	Green River, Munfordville, Hart Co., Kentucky, USA	5	2	2	13	302909	JF304801, 304804, 304807
	Cahaba River, Marvel, Bibb Co., Alabama, USA	4	2	2	6	301169	JF304805, 304808
*Ancylus fluviatilis*	Abhainn an Chnoic, Indreabhán, Galway, Ireland		2			300232	JF304802

Table includes taxonomic identities, sampling locations, number of individuals sequenced per molecular marker, number of individuals processed for morphometric analysis, museum reference numbers and GenBank accession numbers.

Lateral (from the left side) and dorsal views of each individual shell were photographed using a Leica DFC320 digital camera system and processed with Image-Pro Discovery 5.1. These individual profiles were employed for geometric morphometric analyses. The general shape of a limpet shell is quite simple and contains relatively few informative homologous points. To better document shell shape variation, we used landmarks to indicate homologous points on the shell, and also adopted semi-landmarks [64] to represent the outline of the shell. The general framework and principles of landmark-based geometric morphometrics are described in [65].

Shell landmarks and semi-landmarks were digitized using tpsDIG 2.16 [66], recorded in X-Y coordinates format. Three landmarks were placed respectively on the top of the apex, at the central point of anterior aperture margin, and at the central point of posterior aperture margin. Sixty semi-landmarks were placed at equal intervals along the outline of the shell profile. Semi-landmarks were later aligned using the bending energy criterion [64] in tpsRelw 1.49 [66] to remove tangential variation, so that even if each point along the curve may not be homologous among individuals, the curves they form are. Shape coordinates of all individuals were then superimposed using the generalized least squares (GLS) procrustes method [67] with CoordGen6 [68], in order to remove variation caused by differences in shell size, position and image angle [65]. Within-group allometric variation was also removed to reduce ontogenetic effects.

Severe sampling constraints restricted the type of morphometric analyses we could perform on our genetically characterized study populations. For instance, it was not possible to perform discriminant analyses, such as canonical variates analysis (CVA), because they require the sample size to be much larger than the number of variables in order to produce interpretable results. Therefore, a principal component analysis (PCA) was performed using PCAGene6 [68] based on the partial warps scores computed with the same software (Fig. 3). Mean shapes of the three groups – Green River *Rhodacmea elatior*, Cahaba *R. elatior* and apically-intact *R. filosa* (2 Choccolocco Creek specimens combined with 8 Tallaseehatchee Creek limpets) – were graphed (Fig. 3A). In addition, a PCA deformation graph (Fig. 3B) was generated to visually represent changes in shape among groups along the first principal component (PC1).

### Molecular and Phylogenetic Methods

DNA was extracted and target fragments of three genes – 761 nt of nuclear ribosomal large-subunit (28S), 660 nt (aligned) of nuclear ribosomal second internal transcribed spacer (ITS-2) and 654 nt of mitochondrial mt cytochrome oxidase I (COI) – were amplified and directly sequenced (both strands) using the molecular techniques and primer pairs detailed previously [24]. The resulting chromatograms were edited using Sequencher 4.9 (Gene Codes Corporation, Ann Arbor, MI). Edited COI sequences were assembled using Se-Al v2.0a11 [69] with sequences of Ancylinae from GenBank. ITS-2 sequences were aligned using ClustalX [70] and refined manually using Se-Al. Novel genotypes have been deposited in GenBank (Table 1).

For the COI dataset, maximum parsimony (MP), maximum likelihood (ML) and Bayesian analyses were performed using Laevapecini haplotypes as outgroups. MP searches were heuristic using PAUP* 4.0b10 [71] with 100 random stepwise additions, and tree-bisection-reconnection (TBR) branch-swapping. Bootstrap [72] branch support (BS) levels were estimated with 100 replicates, 10 random additions each. ML analysis was performed using PAUP* under TrN+I+G, the best-fit model of nucleotide substitution selected by the Akaike Information Criterion in Modeltest 3.06 [73]. The MP tree was used as the starting tree with the likelihood parameters found in Modeltest and the TBR branch-swapping algorithm. Bootstrap support values were generated using a fast-heuristic search with 100 replicates. Bayesian search was run for 1×10^6^ generations in MrBayes 3.0b4 [74] using the GTR+I+G model. Posterior probabilities were calculated by creating a majority-rule consensus for every 100^th^ tree after burn-in of the first 1000 trees using PAUP*. For ITS-2 sequences, MP analysis was conducted with gaps treated as new characters and *Ancylus fluviatilis* employed as the outgroup.
